# How to select the correct patients for home phototherapy of neonatal hyperbilirubinaemia—a retrospective population-based implementation study

**DOI:** 10.1007/s00431-025-06353-9

**Published:** 2025-07-31

**Authors:** Miriam Pettersson, Cecilia Lai, Andreas Ohlin

**Affiliations:** 1 Department of Paediatrics, Faculty of Medicine and Health, Örebro University Hospital, S-701 85 Örebro, Sweden; 2 Värnamo Hospital, Värnamo, Sweden

**Keywords:** Neonatal hyperbilirubinemia, Home phototherapy, Jaundice, Phototherapy

## Abstract

To examine how many patients received home phototherapy and describe the major reasons for admission to hospital for phototherapy. The study was performed as a retrospective population-based observational study. All newborns at Örebro University Hospital born in October 2019–March 2023 ≥ gestational week 36 + 0 and having performed phototherapy were included. The primary outcome was the proportion of newborns receiving hospital vs home phototherapy. Secondary outcomes were the reasons for not performing home phototherapy. Further secondary outcomes were the number of readmissions (failed home treatments) and the number of infants with bilirubin > 425 µmol/L. In total, 492 patients were included. Of these, 256 (52%) received home phototherapy (180 exclusively, and 76 for part of the treatment); 236 (48%) received phototherapy exclusively in hospital. Among the 302 (236 + 66) patients in whom home phototherapy was not primarily performed, the most common reasons were haemolytic disease (35%) and the need for the use of multiple phototherapy devices (26%). In total, 14% received hospital phototherapy for various other reasons, such as concerns regarding breastfeeding or poor weight gain of the baby.

* Conclusion*: In this retrospective study, we found that approximately 50% of full-term infants with neonatal hyperbilirubinaemia were suitable for home phototherapy. The most common reasons for not receiving home phototherapy in our population were haemolytic disease and severe hyperbilirubinaemia. These results indicate what may be expected when planning to start a home phototherapy programme.

**Table:** 

**What is Known:** • *Phototherapy at home is considered a safe alternative to hospital treatment for well-selected patients, but the proportion of suitable patients has not been studied*.	
**What is New:** • *This study shows that approximately 50% of all full-term newborns with hyperbilirubinaemia can safely be treated at home*. • *The most common contraindications to home phototherapy are haemolytic disease and need for intensive phototherapy*.

**What is Known:**

• *Phototherapy at home is considered a safe alternative to hospital treatment for well-selected patients, but the proportion of suitable patients has not been studied*.

**What is New:**

• *This study shows that approximately 50% of all full-term newborns with hyperbilirubinaemia can safely be treated at home*.

• *The most common contraindications to home phototherapy are haemolytic disease and need for intensive phototherapy*.

## Introduction

Hyperbilirubinaemia is a condition that affects over half of all newborns and that is part of the transition to life. In most cases, the hyperbilirubinaemia will disappear during the newborn’s first weeks of life. However, about 3% of newborns need treatment. In most cases, treatment can be successfully performed using phototherapy which usually is administered in a hospital setting [1–3].

Home phototherapy for neonatal hyperbilirubinaemia has been made possible through the development of easy-to-handle fibre optic equipment. The use of this equipment has been studied and reported to be effective [4–10]. However, starting home phototherapy needs a well-thought-out routine to enhance the safety and effectiveness of the treatment when parents handle the equipment at home. It has been established that home phototherapy is a good alternative to hospital treatment for a well-selected group of patients [9, 11–14]. In the updated guidelines on neonatal hyperbilirubinaemia by the American Academy of Paediatrics (AAP), home phototherapy is presented as an alternative to hospital treatment [1]. However, the British National Institute for Health and Care Excellence (NICE) guidelines from 2023 still fail to mention this treatment option [3].

There are different criteria for establishing which newborns may be considered for the home treatment. Some of these include that the newborns must be otherwise healthy, be term or near term, have a certain level of bilirubin at the start of treatment, and have parents who are willing to perform the treatment at home.

It has also been shown in previous publications that practising home phototherapy lowers parental stress and increases bonding between the parents and the newborn [15]. In addition, studies show that home treatment is cost-effective compared with phototherapy performed in hospital [16] and that parents in general feel secure in handling the treatment [17, 18]. In conclusion, there are several good reasons to consider starting a home phototherapy programme. However, many hospitals still do not offer this alternative to their patients [19].

Even if the criteria for home phototherapy are fairly well described, there is a lack of knowledge on how many patients are suitable for home phototherapy. To answer this question, we designed this retrospective study with the primary aim to examine how many patients received home phototherapy and describe the major reasons for not choosing home phototherapy and instead admitting the patient to the hospital.

## Methods

This is a retrospective observational study; all newborns at Örebro University Hospital born between October 2019 and March 2023 at gestational week ≥ 36 + 0 and having performed phototherapy were included. Local treatment guidelines were established in 2019 after a randomised controlled trial (RCT) on home treatment was finalised [11]. To be eligible for home phototherapy, the newborn must be born at gestational age ≥ 36 weeks and be at least 48 h old, have a bilirubin level of ≤ 400 µmol/L, and be otherwise healthy (adequate weight gain, etc.). Most of the otherwise healthy newborns were discharged from the maternity ward between 6 and 48 h after birth; the newborn then returned for outpatient revisits to check bilirubin and weight gain. All newborns were screened with transcutaneous bilirubin at least once before discharge. The direct antiglobulin test (DAT) is performed on all newborns that need phototherapy before a decision is made on whether the newborn is eligible for home treatment or not. If the newborn has a haemolytic disease, treatment must be initiated in hospital but can be completed at home. Home phototherapy is always optional for the parents. To perform it, parents receive oral and written instructions before starting the treatment. The parents also receive information about neonatal hyperbilirubinemia and why it is important to perform the treatment. They have around-the-clock telephone support (technical and clinical) from the nurses at the neonatal ward. During home phototherapy, all families return to the hospital once daily for bilirubin testing and weight check-ups.

All patients performing home phototherapy used the Bilisoft (GE healthcare, Chicago, IL, USA) which is a fibreoptic device with LED light (spectral irradiance 35 +/− 5 µW/cm^2^/nm, bandwidth 445–470 nm, blue light.). The patients who received phototherapy in the hospital used the Bilisoft or the Medela phototherapy lamp, an overhead lamp placed 25 cm above the patient (Medela, Baar, Switzerland) (spectral irradiance 30 µW/cm^2^/nm, bandwidth 425–475 nm, fluorescent blue light). If the need for more intensive phototherapy arose (by the use of multiple light sources or another phototherapy device than the Bilisoft) during home phototherapy, the patients had to be admitted to the hospital. The patients were identified by searching for International Classification of Diseases and Health-related Problems (ICD-10) codes for jaundice treated with phototherapy (P59) and haemolytic disease (P55). In Örebro, diagnostic codes for newborn conditions are recorded in two different medical record systems and in the Swedish Neonatal Quality Register. To find all the newborns with ICD codes P59 and P55, all three of these systems were searched. Örebro University Hospital is the only hospital in the area that offers maternal delivery and neonatal care, meaning that all or almost all cases of neonatal hyperbilirubinaemia among the population of Örebro County were included in the study. The number of births during the study period was obtained from Statistics Sweden (SCB).

The primary aim was to determine to what extent hospital or home phototherapy was performed. Secondary aims were to study how many of the newborns would have been eligible for home phototherapy following the local treatment guidelines and, in cases where the newborn was treated in hospital, to establish why phototherapy was not administered at home. Further secondary aims were to analyse the number of readmissions (failed home treatments) and the number of infants with bilirubin levels exceeding 425 µmol/L.

Demographic and medical data were recorded in an Excel file that was stored on the hospital’s research server, according to Region Örebro County’s routines for storing research data. Cases of missing data were omitted from the analysis. The study was approved by the regional ethical review board in Uppsala, Sweden (registration number 2023–06406-01).

## Results

**Fig. 1:**
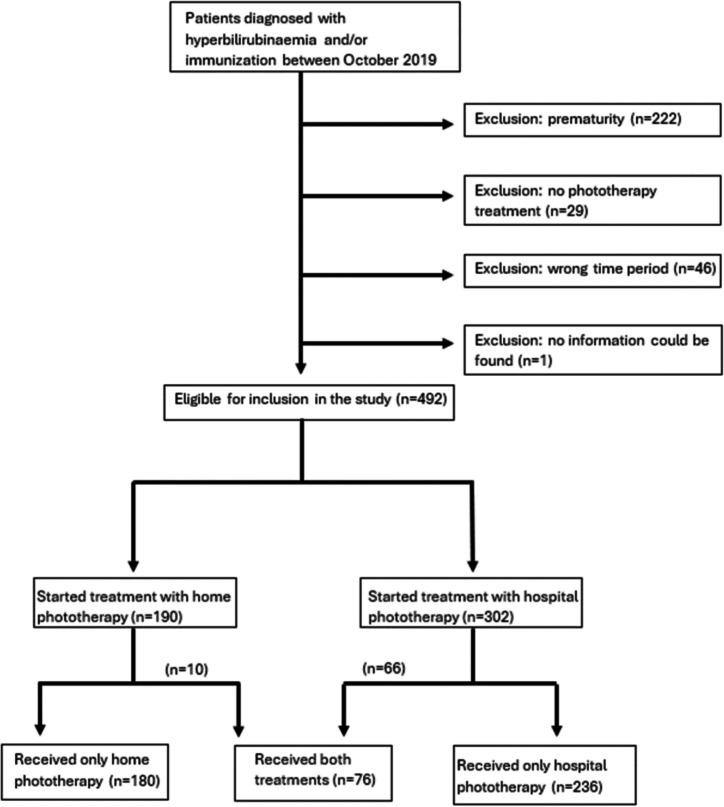
Flow diagram of patients included/excluded in the study and the study groups

Altogether, 790 records matched the ICD codes for haemolytic disease and jaundice and 492 patients were eligible for inclusion in the study (see Fig. 1).

During the study period, 11,027 children were born in Örebro County and there was an incidence of 4.5% of hyperbilirubinaemia in infants born at ≥ 36 weeks’ gestation. Characteristics of the study group are presented in Table 1.

**Table 1:** Characteristics of the study group

Categories	Home phototherapy (*n* = 190)	Hospital phototherapy (*n* = 302)	All included patients (*n* = 492)
Sex (male/female), *n*	117/73	166/136	283/209
Delivery (V/C/I), *n*	137/18/35	215/33/54	352/51/89
Gestational age at inclusion, mean (SD)	38 wk + 5 d (10 d)	39 wk + 0 d (10 d)	38 wk + 6 d (10 d)
Birth weight, g, mean (SD)	3,414 (466)	3,455 (505)	3,439 (490)
Weight at admission, g, mean (SD)	3,241 (438)	3,303 (533)	3,279 (499)
Age at admission, hrs, mean (SD)	102 (40)	69 (45)	81 (46)
Bilirubin at admission, µmol/L, mean (SD)	348 (24)	307 (95)	323 (78)
Haemoglobin at admission, g/L, mean (SD)	195 (19)	186 (28)	191 (24)
Bilirubin level ≥ 425 µmol/L at initiation of phototherapy, n	0	21	21
Bilirubin level ≥ 425 µmol/L after initiating phototherapy, n	2	3	5

*C*, Caesarean section; *I*, instrumental vaginal delivery; *SD*, standard deviation; *V*, vaginal delivery

Of the 492 included patients, 256 (52%) received home phototherapy (180 exclusively, and 76 for only part of the treatment). A total of 236 (48%) received phototherapy in hospital. Of the 180 patients who received home phototherapy only, seven patients were direct antiglobulin test (DAT)-positive. None of these patients developed a rapid increase in serum bilirubin normally associated with haemolytic disease. The patients started home phototherapy at a mean age of 102 h (range 74–129 h) after birth. A comparison of the two treatment groups showed that the patients primarily treated in hospital were younger at treatment initiation and had lower levels of bilirubin combined with a higher incidence of DAT positivity. Of the patients included in the study, all patients with haemolytic disease were caused by blood group incompatibility, and there were no cases of G6PD or spherocytosis.

In the 302 patients in whom home phototherapy was not performed, or not performed primarily (Table 2), the most common reasons were haemolytic disease due to blood group incompatibility (35%) and that the doctor in charge decided that there was a need for more intensive phototherapy with the use of multiple phototherapy devices (26%) even if there were no signs of blood group incompatibility. Altogether 14% received hospital phototherapy for various other reasons, such as the mother being admitted to the maternity ward, concerns regarding breastfeeding, and poor weight gain of the baby. Other common diagnoses in the newborns that contraindicated home phototherapy were most commonly hypoglycaemia and transient tachypnea.

**Table 2:** Main reasons for not choosing/receiving home phototherapy (*n* = 302)

Reasons	*n* (%)
Haemolytic disease	106 (35)
Treatment with multiple phototherapy devices	79 (26)
Other reasons*	41 (14)
Other neonatal illness**	34 (11)
Weight loss > 10%	16 (5)
Parents declined	6 (2)
Parents were not asked	1 (0.3)
Unknown	19 (6)

^*^Other reasons: for example, the mother was not ready for discharge, or there were feeding problems

^**^Other neonatal illness: hypoglycaemia, asphyxia, respiratory distress

Ten patients received exchange transfusion, all in the hospital phototherapy group. No patients received intravenous immunoglobulins.

All in all, 24 patients who received hospital phototherapy had a bilirubin level ≥ 425 µmol/L (24.9 mg/dL). Twenty-one of these had ≥ 425 µmol/L before initiating phototherapy and three after initiating phototherapy. In the home phototherapy group, no patient had a bilirubin level ≥ 425 µmol/L (24.9 mg/dL) before initiating treatment but two infants rose above this threshold during treatment and were therefore admitted to hospital for intensive phototherapy with multiple phototherapy devices. In the first case, the newborn started treatment at 65 h of age at a bilirubin level of 374 µmol/L (21.9 mg/dL). By the first follow-up, at 87 h of age, the bilirubin level had increased to 452 µmol/L (26.4 mg/dL). The baby was admitted to hospital and received intensive phototherapy for 2 days before discharge. In the second case, home phototherapy was initiated at 90 h of age at a bilirubin level of 371 µmol/L. By the first check-up, the following day (at 116 h), the parents had only performed 8 h of phototherapy at home, and the bilirubin level had increased to 426 µmol/L (24.9 mg/dL). However, when reviewing the samples for this study, the sample at treatment start showed signs of interference suggesting that the measurement might be incorrect and should be repeated. Unfortunately, this was not done, and the patient was sent home for home phototherapy without a reliable sample. At the check-up, the patient was admitted to hospital and received intensive phototherapy for 1 day. Thereafter, just one phototherapy device was needed for another 2 days before discharge.

Apart from these two described cases, there were nine newborns who discontinued home phototherapy and were readmitted to hospital. One was a case of haemolytic disease due to blood group incompatibility that first received hospital phototherapy and then continued phototherapy at home but was readmitted due to a rise in bilirubin levels during home phototherapy. The remaining eight were readmitted based on the parents’ wishes because they felt insecure about using the phototherapy equipment or because there were feeding problems. This corresponds to a 4.3% readmission rate.

## Discussion

Since the introduction of fibre optic devices, the interest in phototherapy at home has increased substantially. This development was accelerated during the pandemic when there were obvious benefits to keeping patients away from the hospital. It was therefore logical that home therapy was included as an alternative in the 2022 AAP guidelines even if there are still some questions to be answered on this topic [1, 20].

One of the major knowledge gaps that we attempted to address in this study was to define what proportion of all patients with hyperbilirubinaemia are suitable for home phototherapy. To our knowledge, this has never been studied before and is vital information for any administrator who is planning to start a home phototherapy programme at their hospital. The result that 37% of the patients in our study received all their phototherapy at home and an additional 15% received a combination of hospital and home treatment means that such a programme can significantly decrease the number of beds needed for this patient group. Many resources could potentially be saved as home phototherapy is significantly cheaper than hospital treatment [16, 18]. Of course, the numbers may be different in a different setting or country depending on geography, the health care system, and the economic incentives, but this study shows that up to at least 50% of phototherapy patients could potentially be transferred to a home phototherapy programme.

A comparison between the two treatment groups revealed some expected differences. The patients treated in hospital started their treatment earlier and had lower levels of bilirubin at admission but a high incidence of haemolytic disease. This is an effect of the local treatment protocol that stipulated that patients with haemolytic disease or rapidly increasing bilirubin levels should receive treatment in hospital instead of at home.

Adherence to the local treatment protocol also resulted in the two most common reasons for not choosing home phototherapy: haemolytic disease and severe hyperbilirubinaemia with a need for intensive phototherapy using multiple devices. Newborns with these disorders are at high risk and should not be considered for primary home phototherapy. However, there are several examples in this study of newborns with haemolytic disease who safely finished their phototherapy at home once the bilirubin levels had been stabilised in the hospital and there was no longer a need for multiple phototherapy devices.

There was one patient with haemolytic disease in our follow-up who received hospital phototherapy for 1 day and then was discharged to home phototherapy the following day. This patient was readmitted to hospital after 1 day of home phototherapy because of an increase in bilirubin readings during this time. Although the increase was not hazardous, this patient illustrates the importance of not initiating home phototherapy until the rate of increase in bilirubin can be controlled with a single phototherapy device.

Other common reasons for not offering home phototherapy were often more practical, such as the mother not being ready for discharge from hospital or the infant having feeding problems that require 24-h support. These are totally adequate reasons for performing phototherapy in hospital. There will always be some patients and families who need to stay in hospital a little longer. If the hospital has a well-functioning home care programme for mothers, the above reasons concerning maternal readiness and feeding problems can mostly be managed. The mother and infant can go home earlier, and home phototherapy can be offered as an alternative for the newborn infant.

Rising bilirubin during treatment is something that is desirable, but perhaps not always possible to avoid. In this study, it was seen in both treatment groups. Very few patients (only 2/256 patients, corresponding to 0.8%) in the home phototherapy group needed to be readmitted to hospital because of elevated bilirubin levels. This number is quite substantially lower compared with the 4% we found during the RCT in 2016–2019 [11, 15–17]. This may indicate that the centre’s capacity to address the needs of mother and infant and the staff’s ability to inform patients on how to perform home phototherapy (leading to a greater adherence to planned treatment) have improved over time. However, the total readmission rate was 4.3%, which is about the same as previously described in the RCT performed by the same research group. In most of these cases, the readmission was associated with either the parents feeling insecure about handling the equipment or the newborn having issues (for example related to feeding). These reasons may be avoidable partly with clear and understandable information about the equipment and with hands-on advice about handling a newborn, and so on. 24/7 telephone support by nurses is effective and may increase parents’ sense of security at home; this has been previously evaluated in a qualitative study where parents have described a greater sense of security when having the opportunity to call if needed [17]. However, there will most probably always be some parents who do not feel secure at home, and in these cases, the option to return to the hospital should always be available.

The strength of this study is that we used three different sources to identify potential patients and that we performed the study at a centre with long and well-documented experience of performing home phototherapy. In addition, the inclusion and exclusion criteria were well known at the centre since it had participated in the earlier RCT [11, 15–17]. Another strength is the population-based design as the hospital is the only neonatal hospital within an approximately 100-km radius, which has ensured that the vast majority of patients with hyperbilirubinaemia in the population were included in the study. An obvious weakness of the study is the single-centre retrospective design, which limits the size and the generalizability of the study’s findings.

Since the patients in this retrospective study were treated before the new AAP guidelines with separate treatment thresholds for different gestational ages, this principle was not implemented in this study. We are aware of the advantages of such an arrangement, and if separate treatment levels for different gestational ages would have been used during this study, it would probably affect the study results and might lead to a variation in the number of eligible patients for home phototherapy in different gestational ages.

Another weakness is that at the hospital where this retrospective study was performed only Bilisoft was used for home phototherapy, which means there was no means to increase the intensity of phototherapy at home with either added phototherapy devices or with a single device but with a higher irradiance. If another fibre optic device with a higher irradiance had been available to use for home phototherapy, it could reduce the need for hospital phototherapy further. Also, the decision that the patient needed intensive phototherapy with multiple devices was decided by the doctor in charge at the time and was a subjective clinical decision not based on a specific serum bilirubin level. This may have had an impact on the size of the group of patients who did not receive home phototherapy. A more distinct treatment guideline on when to escalate care may make the decision to use added light sources more objective.

## Conclusion

In this single-centre, population-based retrospective study, we found that home phototherapy is suitable for approximately 50% of full-term infants with neonatal hyperbilirubinaemia. The most common reasons for not receiving home phototherapy were haemolytic disease and severe hyperbilirubinaemia, which required intensive phototherapy. These results indicate what can be expected from home therapy and as such may be useful to anybody planning to start a home phototherapy programme. However, local factors such as economics, geography, and administrative routines may yield different results.

## Data Availability

The data that support the findings of this study are openly available in figshare at Data from homephototherapy implemenation—retrospective population-based study, reference number (10.6084/m9.figshare.29117636).
